# Tumor organoids for testing metabolic flexibility in precision oncology

**DOI:** 10.3389/fonc.2026.1902688

**Published:** 2026-09-22

**Authors:** Di Hao, Yijie Ren, Hui Zhang

**Affiliations:** 1 Graduate School of China Academy of Chinese Medical Sciences, Suzhou, China; 2 Crown Bioscience Inc., San Diego, CA, United States; 3 Affiliated Hangzhou First People’s Hospital, School of Medicine, Westlake University, Hangzhou, China

**Keywords:** cancer metabolism, metabolic flexibility, metabolic heterogeneity, patient-derived models, precision oncology, tumor microenvironment, tumor organoids

## Abstract

Tumors adapt their metabolism to fluctuating nutrients, hypoxia, stromal interactions, immune pressure, and treatment. Tumor organoids are attractive systems in which to test this flexibility, but existing reviews have largely emphasized organoid applications, physiologic culture conditions or metabolomics workflows rather than the strength of evidence behind individual metabolic claims. Here, we address a narrower problem: which metabolic properties of a patient tumor are directly retained or recreated in organoid culture? We evaluate organoid evidence using three levels of support: direct metabolic measurements and perturbation in organoids, biological mechanisms established in other models that require organoid validation, and translational associations that require prospective clinical testing. Recent studies provide direct advances in pancreatic cancer organoids, including subtype-specific oxidative and glycolytic states, KRAS-associated cholesterol dependence and hypoxia-driven invasion, and in gastric and colorectal models where engineered co-cultures expose niche-dependent lipid or immune responses. These findings also expose a central gap: matched benchmarking of organoid metabolic flux and spatial heterogeneity against native tumors remains uncommon. We propose a validation framework based on physiologically specified environments, spatial and isotope-resolved measurements, multi-compartment co-culture and prospective linkage of functional readouts to patient outcomes. Tumor organoids can therefore advance cancer metabolism most credibly as tested, context-specific models rather than assumed replicas of metabolic flexibility *in vivo*.

## 1 Introduction

Cancer metabolism is dynamic rather than fixed. Malignant cells alter nutrient acquisition, redox balance, biosynthesis and intercellular exchange as oxygen, nutrient supply, tissue context and treatment change (1, 2). The experimental challenge is consequently not only to identify a metabolic pathway, but also to determine whether its dependence persists under conditions that resemble a patient’s tumor.

Conventional monolayer cultures remain powerful for perturbation and screening, yet abundant media and ambient oxygen can suppress dependencies that arise in resource-limited tumors. Physiological media have already shown that changing extracellular nutrient levels can rewire measured cancer-cell metabolism (3, 4). Three-dimensional tumor organoids add architecture, patient-specific genotype and selected lineage features to this experimental landscape (5, 6). These attributes make them promising for studying metabolic flexibility, but none guarantees metabolic fidelity. **Table 1** positions two-dimensional cultures, tumor organoids and *in vivo* models against the metabolic questions each is best suited to answer.

**Table 1:** Model choice for cancer-metabolism and metabolic-drug questions.

Model system	Best use	Main advantage	Main limitation	Role
2D cancer cell line	High-throughput perturbation and target discovery	Fast, inexpensive and genetically tractable	Flat architecture; weak oxygen, nutrient and matrix gradients	Screen
Tumor organoid	Patient-matched testing of context-dependent metabolic states	Human genotype, 3D gradients, matrix control and paired perturbation	Requires benchmarking; incomplete vasculature, immunity and pharmacokinetics	Rank
*In vivo* model	Systemic exposure, toxicity, immune and organism-level validation	Captures whole-body drug handling and selected microenvironmental effects	Lower throughput; species and engraftment context can limit human translation	Confirm

This table positions each experimental system within a staged metabolism-targeting drug-discovery workflow. Two-dimensional cancer cell lines are best suited to rapid first-pass perturbation, tumor organoids to patient-matched mechanism ranking under controllable three-dimensional oxygen, nutrient and matrix cues, and *in vivo* models to systemic confirmation rather than high-throughput screening.

A recurring weakness in this field is that metabolic findings from tumor tissue, mouse models or cell lines are often presented adjacent to organoid technology as if they were already demonstrated in organoids. This conflates a biological rationale with a validated model application. The distinction matters because metabolic phenotype depends on medium, extracellular matrix, oxygen transfer, cellular composition and treatment history, each of which can differ between a culture and its source tumor.

The novelty of this Review is therefore not a claim that organoids and cancer metabolism are untouched topics. The field has already recognized the promise of physiologic media, three-dimensional culture, organoid metabolomics and patient-derived drug testing. The unmet need is an evidence boundary: a practical way to decide when an organoid study has tested metabolic flexibility itself, and when it has only provided a plausible experimental setting for biology established elsewhere.

Recent primary studies now permit a more exact account. Patient-derived tumor organoids (PDOs), three-dimensional cultures established directly from patient tumor tissue, have been used in pancreatic cancer to measure subtype-associated metabolic states, hypoxia-induced invasion and therapeutic vulnerabilities (7–10). Engineered gastric organoid co-cultures have uncovered niche-induced lipid vulnerabilities centered on lanosterol synthase and acetyl-CoA carboxylase (11), while colorectal organoid and natural killer cell co-culture has connected organoid exposure to hypoxia-associated immune programs (12). These studies strengthen the case for organoids, but they also reveal that direct metabolic validation is concentrated in a small number of settings.

This Review therefore asks a narrower and more useful question than whether organoids are generally superior models: how can tumor organoids be used to test metabolic flexibility with claims proportional to their evidence? We distinguish direct organoid metabolic evidence from mechanisms awaiting validation in organoids, identify recent advances that change the field, and propose the measurements required to connect organoid phenotype to native tumor metabolism and clinical response. **Figure 1** previews this evidence logic. **Figure 2** maps the organ-specific metabolic constraints that recur across the tumor types considered here.

**Figure 1:**
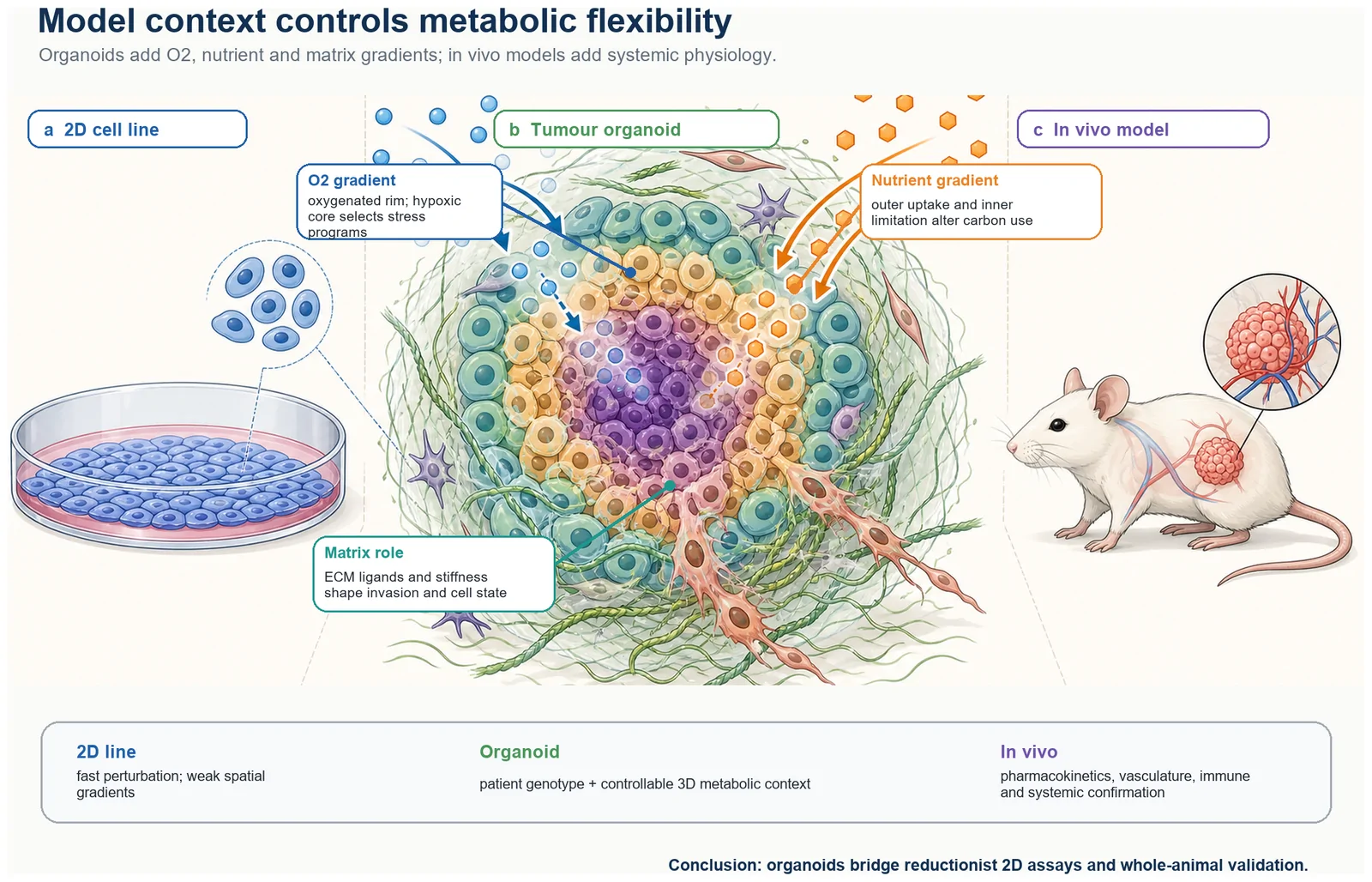
Model context for testing metabolic flexibility. Two-dimensional cell lines support fast perturbation but minimize spatial gradients. Tumor organoids add controllable oxygen, nutrient and matrix gradients that can reveal adaptive metabolic states. *In vivo* mouse models provide systemic pharmacokinetic, vascular, immune and organism-level confirmation, but with lower throughput and species-context limitations.

**Figure 2:**
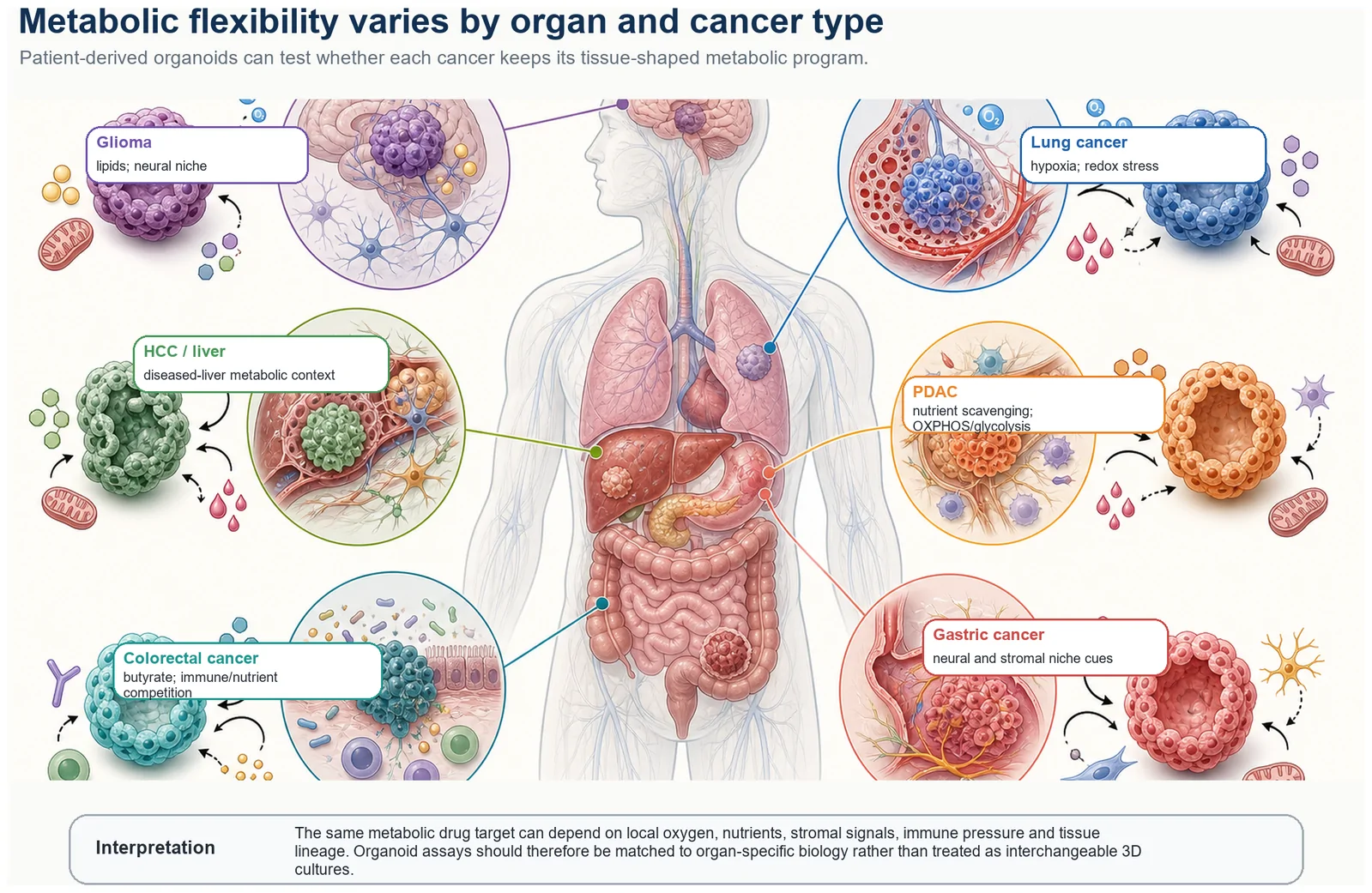
Organ- and cancer-type-specific metabolic flexibility in tumor organoids. Different cancers encounter distinct metabolic constraints, including hypoxia and redox stress in lung cancer, nutrient scavenging and glycolytic or oxidative states in pancreatic cancer, diseased-liver metabolic context in hepatocellular carcinoma, microbiome- and immune-linked metabolism in colorectal cancer, neural or stromal niche cues in gastric cancer, and lipid-neural programs in glioma. The schematic emphasizes that organoid metabolic assays should be matched to the organ context and claim being tested.

## 2 An evidence framework for organoid cancer metabolism

### 2.1 Metabolic fidelity must be measured

Organoid architecture can generate diffusion-limited oxygen and nutrient gradients, as established in three-dimensional tumor models (13, 14). New imaging approaches strengthen this capability: fluorescence lifetime imaging has revealed heterogeneous metabolic states in colorectal cancer three-dimensional cultures, and oxygen-sensitive nanoparticle imaging has resolved oxygenation differences in spheroids (15, 16). Gas-modulating microcapsules further enable controlled perturbation of hypoxic exposure (17). These tools show that gradients can be engineered and detected; they do not show by themselves that a specific organoid reproduces the gradients or metabolic fluxes of its source tumor.

The distinction between metabolic state and metabolic flux is central. A transcriptional signature, metabolite abundance profile or optical redox signal may nominate a pathway, but isotope tracing or functional rescue is generally required to determine how carbon or nitrogen is used. Spatial single-cell isotope tracing recently resolved heterogeneity in fatty-acid synthesis and uptake in cancer models and tumor-bearing brain tissue (18). Applying such flux-sensitive approaches to matched tumor-organoid pairs would address a current missing link: whether organoids preserve the spatial metabolic programs that motivate their use.

### 2.2 Three levels of evidence

To keep claims calibrated, we use three evidence levels. Level 1 comprises direct organoid evidence: a metabolic phenotype or metabolically induced behavior is measured in a tumor organoid and tested by perturbation. Level 2 comprises candidate biology: a tumor metabolic dependency is established in patient material, cell lines or animal models, and an organoid system offers a plausible but not yet validated testbed. Level 3 comprises translation: an organoid readout predicts treatment response or supports a drug-development decision, ideally in a prospective design. **Table 2** summarizes how these levels should constrain interpretation.

**Table 2:** Three levels of evidence for metabolic claims in tumor organoids.

Evidence level	Definition	Minimum required experiment	Claim that is supported	Claim that is not supported
Level 1 Direct organoid evidence	A metabolic phenotype or metabolically induced behavior is measured in a tumor organoid and tested by perturbation	Metabolic readout in the organoid, plus a perturbation that moves that readout in the predicted direction under stated culture conditions	This organoid displays, and depends on, the stated metabolic process under the stated conditions	That the source tumor shares the phenotype, or that the phenotype predicts patient response
Level 2 Candidate biology	A dependency established in patient material, cell lines or animal models, for which an organoid is a plausible but not yet tested system	No organoid experiment yet; the mechanism rests on non-organoid systems	This is a high-priority, well-motivated hypothesis for organoid testing	That organoids have demonstrated the dependency, or that organoid technology supports the mechanism
Level 3 Translation	An organoid readout predicts treatment response or supports a drug-development decision	Prospectively specified assay with predefined outcome, thresholds and analysis, ideally multicenter	The assay has defined predictive performance for the tested tumor type, treatment and endpoint	Generalization beyond the tested tumor type, treatment, endpoint or line of therapy

This table converts the three evidence levels used throughout the Review into operational criteria. The final column is included because most overstatement in this field arises not from asserting too much about an experiment that was performed, but from extending a valid result to a claim the design cannot support. Levels describe the evidence supporting a specific claim, not the quality or importance of a study; a single paper may contribute Level 1 evidence for one claim and Level 2 evidence for another.

**Figure 1** summarizes the claim-evidence map used throughout the Review. The schematic separates microenvironmental inputs, organoid assay design, metabolic readouts and permitted conclusions. It is intended as a reviewer-facing guardrail: an organoid may test a defined metabolic response without proving that it recreates whole-tumor metabolism.

This hierarchy prevents two symmetrical errors. It avoids dismissing organoid studies because no model captures the full tumor microenvironment, and it avoids labeling a biological mechanism as an organoid finding when the required organoid experiment has not been reported. The most informative recent studies move explicitly between levels: they characterize an organoid phenotype, perturb its mechanism, and benchmark the result against patient material or clinical outcomes.

Recent reviews already define the adjacent territory. Physiologic culture reviews have shown why medium, oxygen and microenvironmental context change cancer metabolic readouts (19); a systematic review of organoid metabolomics and lipidomics has emphasized analytical fragility, matrix effects, low biomass and quality control (20); and broad tumor-organoid reviews describe drug discovery, precision therapy and emerging human tumor organoid platforms (21–23). This Review builds on those foundations but uses a different organizing principle: whether each metabolic claim is directly demonstrated in organoids, inferred from non-organoid biology or supported only as a translational association.

The consequence for metabolic writing is specific: the review should not count the number of tumor types for which organoids exist, but rather the number of metabolic questions for which organoids have produced interpretable, independently anchored evidence.

## 3 Direct evidence for metabolic flexibility in tumor organoids

### 3.1 Pancreatic cancer as the most developed test case

Pancreatic ductal adenocarcinoma (PDAC) is a compelling setting for organoid metabolism because its fibrotic and poorly perfused environment imposes nutrient and oxygen stress. Earlier studies established extracellular protein scavenging, macropinocytosis and stellate-cell alanine secretion as nutrient-support mechanisms in PDAC models (24–26). These observations motivate organoid assays, but the most consequential advance is the recent emergence of studies measuring metabolic phenotype directly in pancreatic organoids. **Table 3** summarizes the direct organoid advances discussed in this section and the next, together with the validation gap that each exposes.

**Table 3:** Direct organoid advances and the gap each exposes.

Context	Direct organoid advance	Supported claim	Remaining gap
PDAC isogenic organoids (7)	KRAS-associated cholesterol vulnerability linked to perhexiline response	Defined organoid genotype can expose metabolic targetability	Patient-subtype generalizability and clinical response linkage
PDAC patient-derived organoids (8)	Subtype-specific glycolytic and oxidative phosphorylation profiles	PDO cohorts can reveal metabolic-state diversity	Matched source-tumor flux and larger validation cohorts
PDAC hypoxia organoids (9)	P4HA1-dependent hypoxia-enhanced invasion across 11 models	Organoids can test oxygen-linked adaptive phenotypes	*In vivo* metabolic-state concordance and treatment response
Gastric neuron-tumor organoids (11)	Niche-dependent lipid metabolic vulnerabilities at lanosterol synthase (LSS) and acetyl-CoA carboxylase (ACC)	Defined co-culture can reveal causal metabolic exchange	Which patient contexts require neural interactions
Colorectal organoid and natural killer cell co-culture (12)	Hypoxia- and transforming growth factor beta-associated natural killer cell programs and targetable response	Immune co-culture can expose testable metabolic suppression	Metabolite and flux measurements in both compartments
PDO clinical prediction (83–85)	Prospective feasibility and response associations	Functional response can inform selected precision-oncology decisions	Added value of metabolism-specific readouts

The examples summarize organoid studies that provide direct, interpretable evidence for cancer-metabolism questions. The remaining-gap column defines the next validation boundary for each setting, such as source-tumor concordance, larger cohort testing, *in vivo* linkage, compartment-resolved flux measurement or prospective metabolism-specific clinical readouts.

Duan and colleagues constructed a series of isogenic murine pancreatic organoids that were either wild type or carried common PDAC driver mutations, representing both classical and basal phenotypes, and screened more than 6,000 compounds. They identified perhexiline maleate as preferentially active against Kras-mutant organoids, inhibiting growth and inducing cell death both *in vitro* and *in vivo*. Single-cell RNA sequencing indicated that the cholesterol synthesis pathway, including its key regulator sterol regulatory element-binding protein 2 (SREBP2), was upregulated specifically in KRAS-mutant organoids, and perhexiline reduced SREBP2 expression and reversed the KRAS-driven upregulation of this pathway, with confirmation in primary human PDAC organoids (7). This study provides Level 1 evidence because metabolic pathway nomination, pharmacological perturbation and organoid response are integrated in the same experimental system.

The pharmacological context of this result deserves explicit statement, because it constrains how the finding should be translated. Perhexiline is not a new chemical entity. It is a prophylactic antianginal drug developed in the 1960s and 1970s whose canonical targets are carnitine palmitoyltransferase 1 and 2, and therefore mitochondrial long-chain fatty-acid uptake rather than cholesterol biosynthesis (27). Its clinical use was curtailed by severe hepatotoxicity and peripheral neuropathy, leading to withdrawal from multiple markets; these adverse effects are attributed substantially to drug accumulation in individuals with poor cytochrome P450 2D6 (CYP2D6) metabolizer status, and continued use in the few countries that retain the drug depends on plasma-concentration-guided dosing (27, 28). Two consequences follow for how the organoid result should be read. First, the screen nominates a mechanism, cholesterol biosynthesis dependence in KRAS-mutant PDAC, considerably more convincingly than it nominates perhexiline itself as a deliverable oncology agent. Second, the divergence between the drug’s established carnitine palmitoyltransferase pharmacology and the SREBP2-associated effect measured in organoids is itself an unresolved question rather than a settled mechanism. Target-engagement experiments in organoids under physiologically specified lipid conditions, rather than viability readouts alone, would be needed to establish which mechanism dominates at exposures achievable in patients.

A complementary patient-derived study found that metastatic PDAC organoids of basal-like and classical subtypes differ in glycolytic and oxidative phosphorylation profiles, and the direction of those differences is more instructive than a simple glycolytic-versus-oxidative dichotomy. In the Seahorse glycolytic stress test, basal-like PDOs showed a lower baseline extracellular acidification rate (ECAR) but a higher glycolytic reserve, measured as the difference between glycolytic capacity and baseline glycolysis, which indicates that classical PDOs operate closer to their maximum glycolytic potential. In the mitochondrial stress test the same lines showed higher baseline, maximal and spare capacity oxygen consumption rates (OCR) and higher OCR to ECAR ratios. The OCR difference was eliminated by the mitochondrial pyruvate carrier 1 (MPC1) inhibitor UK-5099, which produced a marked decrease in maximal OCR that was more pronounced in basal-like lines, indicating that the oxidative advantage of basal-like organoids depends on mitochondrial pyruvate import rather than on a generalized increase in respiratory capacity. In parallel 13C-glucose tracing, a basal-like PDO showed lower fractions of some M + 2 metabolites but greater sensitivity to UK-5099-mediated reduction of M + 2 labeling than a classical PDO. Protein-level analyses found lower MPC1 in basal-like cases, and low MPC1 was associated with clinicopathological parameters of tumor aggressiveness (8).

The cohort was limited, comprising three basal-like and five classical lines, so the finding identifies a biologically plausible subtype-associated vulnerability rather than a validated biomarker. Its importance lies in demonstrating how organoid metabolic profiling can be made directly measurable and falsifiable. A specific transporter was nominated, inhibited pharmacologically, and shown to collapse the very phenotype it was proposed to explain, which is a materially stronger form of evidence than a correlative metabolic signature.

Microenvironmental stress has also been tested directly. Across 11 patient-derived PDAC organoid models, hypoxia increased invasion, and perturbation experiments identified prolyl 4-hydroxylase subunit alpha 1 (P4HA1) as necessary for hypoxia-enhanced invasion and sufficient to increase invasion under normoxia (9). Although this is an invasion study rather than a complete metabolic flux analysis, it links a defined microenvironmental constraint to a patient-derived organoid behavior and a manipulable mechanism. Taken together, the three claims developed in this section share a structure worth naming. In each, a pathway was nominated inside the organoid, perturbed inside the organoid, and the phenotype moved in the predicted direction. What none of them establishes is that the organoid reproduces the metabolic organization of the tumor it was derived from.

At a broader translational scale, a pancreatic cancer organoid biobank has linked multi-omic features to therapeutic response and carried a metabolic hypothesis into a clinical trial. Li and colleagues established 260 PDAC organoid lines with extensive multi-omic profiling and drug- and radiation-sensitivity assessment, nominating 6 candidate coding and 35 candidate noncoding drivers, 2,794 multi-omic features associated with drug sensitivity and 322 features associated with radiation sensitivity. Pharmacogenomic analysis showed that chemoresistant organoids were enriched for protein glycosylation and cholesterol metabolism pathways, and statins selectively targeted chemoresistant PDAC organoids, attenuating protein glycosylation, cholesterol levels and the epithelial-to-mesenchymal transition signature. The authors then conducted a single-center, single-arm phase 2 trial (NCT06241352) combining atorvastatin with chemotherapy in advanced pancreatic cancer, in which 26 of 37 patients (70.3%) met the reported response definition of a decrease of more than 20% in tumor markers (10).

This is the clearest current example of a Level 3 claim built on organoid metabolism, and it is equally useful as an illustration of what Level 3 evidence does and does not establish. The organoid-to-trial logic is explicit, and the metabolic hypothesis, cholesterol pathway dependence in chemoresistant disease, converges with the KRAS-associated cholesterol vulnerability identified independently in isogenic organoids (7). That a genotype-driven screen and a resistance-driven screen arrive at the same biosynthetic arm from different directions is the strongest internal indication in this Review that an organoid-detected metabolic dependency is tracking tumor biology rather than an idiosyncrasy of one experimental design. The clinical endpoint, however, was a biochemical tumor-marker decline in a single-arm, single-center design rather than a randomized comparison against chemotherapy alone using radiographic or survival endpoints, and an organoid-derived measure of cholesterol dependence was not itself used prospectively to allocate individual patients to atorvastatin. The trial therefore establishes feasibility and a promising signal; it does not yet establish that an organoid metabolic readout predicts statin benefit for a given patient. Keeping those two statements separate is precisely the function of the evidence hierarchy proposed here.

Together, these studies move PDAC organoids beyond generic drug screening toward an experimentally tractable model of metabolic state, stress response and treatment selection. They also make the remaining gap visible: source-tumor spatial metabolism and organoid metabolic flux are rarely compared within the same patient cohort.

That gap is now experimentally approachable, and the reference maps required for matched benchmarking already exist in the same disease. Integrating single-cell transcriptomics, spatial transcriptomics and spatial metabolomics in pancreatic tumor specimens, Wu and colleagues resolved histologically distinct pathological regions and classified them as hypermetabolic or hypometabolic using single-cell metabolic scoring, while pseudotime trajectory analysis showed that metabolic state shifts along malignant progression rather than remaining uniform across the tumor. The design pairs 10x Visium spatial transcriptomics with airflow-assisted desorption electrospray ionization mass spectrometry imaging across six resected pancreatic tumors, with two further specimens collected after neoadjuvant therapy, and the resulting alterations are spatially restricted rather than diffuse. Polyamine synthesis, indicated by ornithine, putrescine, spermidine and spermine together with raised SAT1, SRM and SMS, localized to malignant regions. *De novo* lipogenesis, driven by acetyl-CoA carboxylase and fatty acid synthase with downstream SCD, FADS and ELOVL family desaturases and elongases, was upregulated in tumor areas. Phospholipid species were compartmentalized between tumor, normal and stromal or immune regions alongside Lands-cycle remodeling enzymes such as LPCAT1. Aberrant communication among cancer cells, macrophages and fibroblasts was also detected, with receptor-ligand pairs co-expressed in malignant regions and correlated with poor prognosis (29). It is these region-level assignments, rather than a whole-tumor metabolite list, that make the study usable as a benchmarking reference, because they define the spatial units against which a derived organoid could be scored.

A decisive next step would couple such tissue maps with organoids derived from the same regions or patients, testing which spatial metabolic programs persist in culture and which require added microenvironmental components.

A rigorous paired design would use freshly profiled tumor tissue to define the reference metabolic state, derive organoids from the same clinical specimen, and then perturb variables such as oxygen, nutrient composition or stromal support. A pathway detected in both patient tissue and organoids, and changed in the predicted direction by a mechanistically relevant perturbation, would represent substantially stronger evidence than either omics similarity or organoid drug sensitivity alone. PDAC is currently the best candidate disease in which to establish such a validation pipeline because both organoid perturbation studies and spatial tumor maps are now available.

### 3.2 Niche-induced metabolic vulnerability beyond pancreatic cancer

Recent work in gastric cancer demonstrates why epithelial-only organoids can miss a relevant metabolic dependency, and it names the metabolic nodes involved. Multi-tiered CRISPR screens in a combined enteric neuron and gastric tumor organoid system revealed *in vivo*-relevant metabolic dependencies and synthetic lethal pairs that were recoverable in tumor organoids but not in immortalized cell lines or even three-dimensional spheroids. The screens converged on two lipid-metabolic enzymes: lanosterol synthase (LSS), which commits the post-squalene arm of cholesterol biosynthesis, and acetyl-CoA carboxylase (ACC), which catalyzes the rate-limiting carboxylation of acetyl-CoA to malonyl-CoA in *de novo* fatty-acid synthesis. Inhibitors of both enzymes showed nanomolar half-maximal inhibitory concentrations across diverse human gastric cancer organoids resistant to first-line treatments and impeded xenografted tumor growth in mice (11).

Three features of that study bear directly on the argument developed here. Gastric cancer organoids and *in vivo* tumors exhibited lipid metabolic adaptations that were not seen in two-dimensional culture, so the dependency is a property of the model context and not only of the genotype. Enteric neurons modulated lipid metabolism in tumor organoids and altered drug sensitivity by up to two orders of magnitude, which is a larger effect than most genotype-based stratifications deliver. A neuron-cocultured CRISPR screen further showed that acetyl-CoA carboxylase expression determines the efficacy of lanosterol synthase inhibition, meaning that the niche altered not only the magnitude of response but the epistasis between two druggable lipid enzymes. The study is therefore important not because every gastric tumor requires a neural model, but because it shows that a defined niche component can change the actionable metabolic phenotype. Organoid complexity is valuable when it is linked to a measurable question, not when complexity is added indiscriminately.

It is worth noting a convergence across these otherwise unrelated systems. KRAS-mutant pancreatic organoids implicate SREBP2-driven cholesterol synthesis (7), chemoresistant pancreatic organoids are enriched for cholesterol metabolism and respond to statins (10), and gastric tumor organoids depend on lanosterol synthase in the same biosynthetic arm (11). Sterol and lipid biosynthesis is thus emerging as a recurrent organoid-detected vulnerability across gastrointestinal cancers. Whether this reflects shared tumor biology, or instead a shared property of organoid culture conditions such as lipid-restricted defined media, is an open question that matched tissue benchmarking would resolve.

Colorectal cancer provides a related example at the interface of metabolism and immunity. In a 2025 study, genetically diverse colorectal patient-derived organoids exposed primary natural killer cells to hypoxia- and transforming growth factor beta-associated programs resembling signatures in patient tumor immune cells; targeting hypoxia-inducible factor or transforming growth factor beta receptor signaling enhanced selected functional responses (12). This provides direct organoid evidence for a stress-linked immune interaction, but not a complete map of nutrient competition or metabolite exchange. Future experiments should pair immune phenotype with measurements of oxygen, lactate, amino-acid availability and tumor-cell metabolic flux.

For colorectal epithelium itself, butyrate remains an instructive metabolic exposure: whether it is oxidized or accumulates as a histone deacetylase inhibitor depends on malignant metabolic state (30). Organoids offer a controlled route to test inter-patient variation in this response, but robust conclusions will require defined luminal metabolite exposure, isotope or respiration readouts and confirmation that growth effects represent the proposed mechanism rather than nonspecific stress.

These gastrointestinal examples expose a gap overlooked by a simple organ-by-organ review: metabolic flexibility is frequently produced by interaction with a niche rather than by the tumor epithelium alone. Yet niche reconstruction should be selective. A neuron, immune-cell or microbial metabolite component belongs in an organoid experiment only when the study tests a defined causal exchange, controls for its addition and removal, and measures a metabolic consequence alongside growth or killing. Otherwise, a more complex culture can add realism in appearance while reducing interpretability.

## 4 Metabolic biology awaiting direct organoid validation

### 4.1 Lung cancer

In lung adenocarcinoma, metabolic mechanisms are biologically compelling but direct organoid validation remains incomplete. KRAS-driven tumors with liver kinase B1 (LKB1) loss depend on carbamoyl phosphate synthetase 1 (CPS1) to maintain pyrimidine pools, and epidermal growth factor receptor (EGFR) mutant tumors can show serine-biosynthesis vulnerabilities during erlotinib resistance (31, 32). Organoid systems representing molecular subtypes have been developed (33), and hypoxia has been reported to alter growth, stemness and cluster migration in lung tumor organoids (34). Nonetheless, the literature does not yet establish that genotype-specific pyrimidine or serine flux dependencies are retained across patient-derived lung organoid cohorts. These should be presented as high-priority tests rather than current organoid achievements.

A tractable lung validation study would derive organoids from tumors with defined KRAS, LKB1 and epidermal growth factor receptor status, retain lineage-marker assessment, and perform isotope tracing during CPS1 or phosphoglycerate dehydrogenase perturbation under physiological nutrient conditions. Comparison with source-tumor metabolite or transcriptional profiles would then test whether the proposed genotype-metabolism relationship survives organoid propagation. Without this step, organoids remain a promising experimental destination for these mechanisms rather than evidence supporting them.

### 4.2 Hepatic and renal cancers

Liver and kidney cancers highlight the need to preserve organ-specific metabolic context. Loss of fructose-1,6-bisphosphatase 1 (FBP1) has been linked to liver tumor evolution in metabolic dysfunction-associated steatohepatitis and to hepatic stellate-cell senescence signaling (35, 36). In clear cell renal cell carcinoma, loss of the same enzyme promotes progression through hypoxia-associated metabolic regulation (37). These findings nominate compelling organoid experiments, including diseased-liver co-culture and lipid- and hypoxia-sensitive renal models. They do not yet demonstrate metabolic fidelity in tumor organoids, and the review accordingly treats them as Level 2 evidence.

The distinction is especially consequential in liver cancer, where chronic metabolic liver disease and non-malignant cell states may drive the phenotype being modeled. A tumor epithelial organoid that omits senescent hepatocytes or stellate-cell signaling cannot be assumed to capture an FBP1-associated niche mechanism. Likewise, a renal organoid study directed at lipid accumulation or oxidative stress needs to show that the cultured model retains clear-cell identity and the relevant hypoxia-associated response before it supports a therapeutic conclusion.

### 4.3 Glioma and metastatic niches

Glioma biology likewise offers mechanistic targets for organoid validation. Lipid metabolism and stress-associated metabolic co-dependencies are prominent in glioma, and glioma stem cells can use polyunsaturated fatty-acid synthesis to support epidermal growth factor receptor signaling (38, 39). Patient-derived glioblastoma organoids preserve selected intratumoral heterogeneity (40), but direct mapping of these lipid flux programs in organoids remains limited. Adding neural tissue interactions may be necessary for some questions, yet it should not be assumed to restore vascular or immune metabolism.

Metastatic adaptation poses an additional boundary. Cancer cells face distinct metabolic conditions during dissemination and organ colonization (41), and limited serine and glycine in the brain can sensitize brain metastasis models to phosphoglycerate dehydrogenase inhibition (42). Organoid-based metastatic niches could test this context dependence, but a therapeutic claim requires comparison with patient metastases or *in vivo* models and an indication of when during metastasis the vulnerability is present.

### 4.4 Breast, ovarian, bladder and head and neck cancers

The concentration of direct organoid metabolic evidence in pancreatic and gastrointestinal cancers reflects the state of the literature rather than a judgement that other malignancies are less metabolically interesting. Outside these settings the evidence falls into two recognizable patterns, and naming them is more useful than an organ-by-organ inventory, because each pattern implies a different next experiment.

In the first pattern the mechanism is well specified but the organoid contribution stops short of causal perturbation. Breast cancer PDOs have been used to examine subtype-associated metabolic adaptation, including altered glucose handling and lipid dependence, and they retain features of the originating tumor that monolayer culture loses, yet systematic flux measurement across annotated cohorts remains sparse (43). Ovarian cancer organoids go further toward mechanism, linking chemoresistance to induction of glucose metabolism through an Aurora-A/SOX8/FOXK1 axis and to glycolytic and angiogenic reprogramming through a Fibrillin-1/VEGFR2/STAT2 axis (44, 45). In both diseases, however, the causal step is frequently carried by parallel cell-line experiments rather than by perturbation inside the organoid, so the organoid supplies the phenotype while another model supplies the mechanism. These studies sit at the boundary between Level 1 and Level 2, and the experiment that would move them is modest: repeat the decisive perturbation in the organoid under specified culture conditions.

The second pattern is the reverse. Bladder and head and neck cancers have well-characterized organoid biobanks that capture tumor evolution and drug response, and organoid drug sensitivity has been shown to track sensitivity *in vivo* (46, 47). Here the models are mature and the clinical anchoring is unusually good, but metabolic phenotyping remains largely descriptive. The limiting resource is not tissue or cohort size but the measurement itself, so the cheapest available route to Level 1 evidence in these diseases is to add flux-sensitive or spatially resolved readouts to collections that already exist rather than to build new ones.

Read together, the two patterns suggest that the pancreatic emphasis of this Review is a statement about where perturbation and measurement have so far been combined, not about which tumors have interesting metabolism. **Table 4** sets out the position across eleven tumor types, including the highest level of metabolic evidence currently available in each and the specific limitation that bounds it.

**Table 4:** Organoid models across cancer types: current metabolic evidence, advantages and limitations.

Cancer type	Representative organoid evidence	Highest current metabolic evidence level	Main advantage for metabolic study	Main limitation for metabolic study
Pancreatic (PDAC)	Isogenic driver-mutation organoids; metastatic PDO subtype panels; hypoxia-invasion models; 260-line multi-omic biobank with statin trial	Level 3	Strong nutrient and oxygen stress biology; matched spatial tissue maps exist in the same disease; perturbation and clinical arms both reported	Cohorts for flux measurement remain small; clinical endpoint to date is a biochemical marker in a single-arm design
Gastric	Enteric neuron and tumor organoid co-culture with multi-tiered CRISPR screens	Level 1	Demonstrates that a defined niche component changes the actionable lipid phenotype and the epistasis between druggable enzymes	Which patient contexts require a neural component is undefined; no matched source-tumor flux comparison
Colorectal	Genetically diverse PDOs in natural killer cell co-culture; butyrate exposure models; prospective response-prediction cohorts	Level 3	Best developed prospective clinical evidence; tractable luminal metabolite exposure	Immune-metabolic studies measure phenotype without nutrient competition or metabolite exchange readouts
Lung	Organoids representing molecular subtypes; hypoxia effects on growth and cluster migration	Level 2	Genotype-defined metabolic hypotheses are unusually well specified, such as CPS1 and phosphoglycerate dehydrogenase dependence	Genotype-specific pyrimidine and serine flux dependence not yet shown to be retained across PDO cohorts
Liver	FBP1-associated tumor evolution from senescent hepatocytes in metabolic dysfunction-associated steatohepatitis; stellate-cell senescence secretome	Level 2	Organ-specific metabolic context is central and well characterized in tissue	Epithelial-only organoids omit the senescent hepatocyte and stellate-cell compartments that may drive the phenotype
Renal (clear cell)	FBP1 loss and hypoxia-associated metabolic regulation in clear cell carcinoma	Level 2	Clear metabolic driver biology with an established hypoxia axis	Retention of clear-cell identity and lipid phenotype in culture is not routinely demonstrated
Glioma	Patient-derived glioblastoma organoids and biobanks preserving intratumoral heterogeneity	Level 2	Preserved intratumoral heterogeneity suits spatially resolved metabolic measurement	Lipid flux programs not directly mapped in organoids; vascular and immune metabolism not restored by neural tissue addition
Breast	PDOs used to examine subtype-associated glucose and lipid adaptation	Level 1 to Level 2	Subtype biology is well defined and PDOs retain features lost in monolayer culture	Systematic flux measurement across annotated PDO cohorts is limited
Ovarian	Chemoresistance linked to glucose metabolism via Aurora-A/SOX8/FOXK1 and to glycolysis via Fibrillin-1/VEGFR2/STAT2	Level 1 to Level 2	Mechanistic chains are explicit and metabolic readouts are obtained in organoids	Causal perturbation is frequently anchored in parallel cell-line experiments rather than in organoids
Bladder	Patient-derived organoid biobanks capturing tumor evolution and drug response	Level 2	Mature, well-annotated biobanks already exist and track *in vivo* drug response	Metabolic phenotyping remains largely descriptive
Head and neck	Patient-derived organoids predicting *in vivo* drug sensitivity; artificial intelligence and digital pathology integration proposed	Level 2	Established link between organoid sensitivity and *in vivo* response provides a validation anchor	Little direct metabolic measurement reported to date

The evidence-level column reports the highest level of metabolic evidence currently available for each tumor type according to the criteria in **Table 2**, not the highest level achievable. It therefore describes the present literature and is expected to change. Advantages and limitations are stated for metabolic study specifically, and differ from the general strengths and weaknesses of organoid models in each disease.

## 5 Measuring metabolism in organoids: analytical limits and standardization

Every claim in the preceding sections rests on a measurement, and the reproducibility of those measurements is not yet standardized across laboratories. Because metabolite pools turn over in seconds to minutes and organoid biomass is small, organoid metabolomics is unusually sensitive to procedural variation. Treating analytical performance as a background technical matter would undermine the evidence hierarchy proposed here, since an irreproducible measurement cannot support a Level 1 claim regardless of how the experiment is framed.

### 5.1 Analytical reproducibility and quality control

Untargeted metabolomics has no single accuracy standard equivalent to a sequencing quality score, and the community has responded by developing consensus quality assurance and quality control practices. Coordinated efforts have defined priority objectives for quality assurance in untargeted studies, surveyed what practitioners actually do, and assessed the reference materials available for benchmarking instrument and workflow performance (48–50). Pooled quality control samples, injected at intervals through an analytical run, remain the principal instrument for detecting signal drift and for separating analytical from biological variance, although their implementation varies considerably between laboratories (51).

Two problems are amplified in organoid work specifically. The first is low biomass. A single organoid well can yield metabolite quantities near the limit of quantification, which compresses dynamic range and inflates the relative contribution of extraction losses. The second is matrix effect. Organoids are embedded in protein-rich basement membrane extract and cultured in media containing growth factors, serum substitutes and high concentrations of defined metabolites, all of which can suppress or enhance ionization and generate carryover signals that are difficult to distinguish from endogenous pools unless explicitly controlled; strategies for quantifying matrix effects in liquid chromatography-tandem mass spectrometry are long established and transfer directly to this setting (52). A systematic review of organoid metabolomics and lipidomics has identified precisely this combination of analytical fragility, matrix effects, low biomass and inconsistent quality control as the dominant methodological limitation of the field (20). Batch-to-batch differences in reagents, extraction solvents and chromatographic columns act on top of these effects, so that studies performed months apart in the same laboratory may not be directly comparable without bridging samples.

### 5.2 Culture-derived variability and normalization

Biological variability in organoid culture is at least as large as analytical variability, and it is more often left unreported. Basement membrane extract derived from murine sarcoma remains the dominant matrix, and its undefined and lot-variable composition introduces growth factor and matrix protein differences that directly affect metabolic phenotype; defined and tissue-derived alternatives exist but are not yet standard (53, 54). Matrix mechanics matter independently of composition. Engineered matrices have shown that stiffness alone drives chemoresistance in patient-derived pancreatic cancer organoids, which means that a metabolic or drug-response difference attributed to genotype may in part reflect the mechanical properties of the gel (55). Passage number, organoid size distribution, seeding density, luminal accumulation of secreted metabolites and the interval since the last medium change all further modify measured metabolic state.

Normalization strategy is the point at which these variables either are or are not brought under control, and it is frequently underspecified. Metabolite abundances and respirometry values normalized to total protein, to DNA content, to cell number or to organoid volume can rank the same samples differently, because organoid preparations differ in the proportion of viable cells, in lumen volume and in matrix carryover, and the choice among available normalization strategies is itself a recognized source of variance in quantitative metabolomics (56). Published protocols for organoid and spheroid bioenergetics address this directly by fixing seeding, dissociation and normalization steps before comparison, and they are better treated as a minimum procedural standard than as optional methodological detail (57, 58). For any comparative metabolic claim, the normalization denominator should be stated, justified, and ideally shown not to alter the direction of the reported effect.

### 5.3 Single-cell and spatially resolved measurement

Bulk measurement of an organoid population averages across precisely the heterogeneity that motivates organoid use, and single-cell and spatial methods are the natural response. Single-cell metabolomics by mass spectrometry can now detect metabolites in individual cells without preselection or labeling, and methods that register mass spectrometry imaging to prior microscopy of the same cells link metabolic state to morphology and to spatial position (59, 60). Spatial single-cell isotope tracing extends this from abundance to flux, which is the measurement class that the arguments in this Review most often require (18).

Spatial transcriptomics and spatial proteomics complete the picture on the regulatory side. Since the introduction of array-based spatial transcriptomics (61), multiplexed imaging platforms have made it possible to quantify tens of proteins with preserved tissue architecture (62, 63), and integrated spatial omics approaches are now standard for describing intratumoral heterogeneity in patient material (64). The integrated spatial study of pancreatic tumors discussed above illustrates what these methods yield when combined (29). Their application to organoids, however, remains far less developed than their application to tissue. Two obstacles are practical rather than conceptual: organoids embedded in hydrogel are difficult to section and register reproducibly, and the small number of cells per organoid limits the statistical power of single-cell metabolic assays. Overcoming these obstacles is a prerequisite for the matched benchmarking proposed here, because benchmarking requires the same measurement class on both sides of the comparison.

### 5.4 Toward inter-laboratory standardization

None of the above requires a new technology. It requires agreement on what is reported. At present, two laboratories can publish organoid metabolic studies of the same tumor type that cannot be compared, because medium formulation, oxygen tension, matrix lot, passage number, normalization denominator and quality control design are each described incompletely or not at all. Reference materials and pooled quality control practices provide a partial solution for the analytical component (50, 51), and dedicated metabolite reference materials now allow inter-laboratory proficiency to be tested directly rather than inferred (65). Ring-trial data show that untargeted assays can reach acceptable inter-laboratory agreement once the protocol is fixed (66). The culture component has no equivalent. A pragmatic path is a minimum reporting set adopted at journal level, following the precedent set for chemical analysis in metabolomics (67), coupled with circulation of shared reference organoid lines and reference metabolite mixtures between laboratories, so that inter-laboratory variance can be measured rather than assumed. **Table 5** sets out the reporting and quality-control items that follow from this section and from the limitations discussed below.

**Table 5:** Reporting and quality-control requirements for organoid metabolic studies.

Domain	Item to report	Why it matters for metabolic interpretation	Minimum standard
Model provenance	Tumor type, subtype, specimen source, prior treatment, establishment success rate and reasons for failure in the parent cohort	Establishment selects for proliferative and culture-adapted cells, so the cohort may under-sample quiescent or stroma-dependent metabolic states	Report establishment numerator and denominator, not only the lines that succeeded
Culture specification	Medium formulation, oxygen tension, matrix type and lot, matrix stiffness where engineered, organoid dimensions, seeding density and interval since medium change	Each variable independently modifies metabolic phenotype; matrix stiffness alone can drive chemoresistance	State all six; justify any that deviate from the cited source protocol
Passage and stability	Passage number at assay, and evidence of phenotype stability across passages for any central claim	Genomic representativeness after passage does not guarantee metabolic stability, because phenotype tracks cell state	Report passage number always; show cross-passage stability for the primary endpoint
Analytical quality control	Pooled quality control sample design and injection interval, reference materials used, extraction protocol, batch structure and bridging samples between batches	Low biomass and protein-rich matrix make organoid metabolomics unusually prone to drift, ion suppression and carryover	Pooled quality control samples through every run; report coefficient of variation for reported features
Matrix and blank controls	Matrix-only and medium-only blanks processed identically to samples	Basement membrane extract and medium components generate signals that can be mistaken for endogenous metabolite pools	Process and report blanks for every batch; exclude features not separable from blank signal
Normalization	Normalization denominator used, with justification, and confirmation that the reported effect direction is preserved under an alternative denominator	Protein, DNA, cell number and organoid volume can rank the same samples differently because preparations differ in viability, lumen volume and matrix carryover	State the denominator; show the primary result is not denominator-dependent
Claim-matched readout	Explicit statement of endpoint class: metabolite abundance, carbon flux, redox state, invasion, immune activity, survival or drug response	An effect on viability alone does not establish a specific metabolic mechanism	Match the assay to the claim; a nutrient-use claim requires a flux-sensitive assay or rescue experiment
Spatial claims	Spatial resolution and registration method for any claim of metabolic heterogeneity	Bulk measurement averages across the heterogeneity that motivates organoid use	A spatial claim requires a spatially resolved measurement, not a bulk measurement with inferred structure
Computational inference	Whether a reported flux was measured or inferred, and the model, inputs and assumptions used for inference	Transcript-derived flux is constrained but not determined by expression, so inferred flux is Level 2 evidence by construction	Label inferred flux as inferred; reserve tracing-grade confidence language for tracing data
Co-culture design	Identity, source and proportion of each added cell type, plus addition and removal controls	Niche components can shift drug sensitivity by orders of magnitude and can alter epistasis between targets	Report the controlled addition and removal experiment, not the co-culture endpoint alone
Benchmarking	Whether source-tumor material was profiled with the same measurement class, and the concordance result	Organoid and source-tumor metabolic concordance is the specific gap this Review identifies	State explicitly when matched benchmarking was not performed
Clinical translation	Prospective or retrospective design, prespecified endpoint and thresholds, turnaround time, and whether the metabolic readout was used to allocate treatment	A promising response signal in a single-arm design does not establish that a metabolic readout predicts benefit	Report whether the organoid readout was used prospectively for allocation; do not describe retrospective association as prediction

These items convert the analytical, culture-derived and model limitations discussed in the Review into reportable and checkable requirements. They are deliberately restricted to information that authors already possess at the time of writing, so that adoption imposes reporting discipline rather than additional experiments. The minimum standard column states what should be considered sufficient for publication of a metabolic claim at the corresponding evidence level.

## 6 Computational integration and AI-assisted metabolic analysis

Computational methods intersect organoid metabolism at three points: inferring metabolic activity that was not directly measured, predicting therapeutic response from organoid data, and integrating organoid readouts with the histological and molecular data already generated in clinical care. Each is developing quickly, and each carries a distinct interpretive risk that maps onto the evidence hierarchy used throughout this Review.

### 6.1 Metabolic network modeling and flux inference

Genome-scale metabolic models and constraint-based methods allow metabolic network states to be estimated from transcriptomic data, and single-cell adaptations now estimate cell-wise flux from single-cell RNA sequencing, including graph neural network implementations that respect network topology (68, 69). Applied to organoids, these methods offer an attractive route to metabolic maps at a resolution and cost that direct flux measurement cannot match. Their limitation is fundamental rather than technical: transcript abundance constrains but does not determine flux, because allosteric regulation, post-translational modification and substrate availability all intervene (70). Inferred flux is therefore Level 2 evidence by construction. It is a powerful hypothesis generator, and it becomes Level 1 evidence only when a nominated flux is confirmed by isotope tracing or by a perturbation that moves the measured phenotype in the predicted direction. Reporting computational flux estimates with the confidence language reserved for tracing data is a category error that the field should avoid.

### 6.2 Machine learning for organoid response prediction

Machine learning applied to organoid drug response has produced credible results where the model is anchored in biological structure rather than fitted to raw feature counts. Network-based machine learning trained on colorectal and bladder organoid models has predicted anti-cancer drug efficacy in patients, indicating that organoid-derived training data can transfer to clinical outcome in the settings tested (71). Image-based deep learning is a complementary and less data-hungry approach, because organoid morphology, growth trajectory and death kinetics are already recorded in ordinary brightfield time-series. Open platforms now track and segment individual organoids over time, and related tools quantify therapeutic effects in organoid and immune-cell co-cultures from time-resolved imaging (72, 73). For cancer metabolism specifically, morphological and kinetic readouts are attractive because they are non-destructive and can be paired with a terminal metabolic measurement in the same well, converting a single endpoint into a trajectory.

### 6.3 Digital pathology and multi-omics integration

Foundation models trained on large whole-slide image collections now support a range of computational pathology tasks and generalize considerably better than task-specific classifiers (74, 75). Their relevance here is that the source tumor of every organoid has already been imaged histologically as part of routine diagnosis, so digital pathology offers an inexpensive bridge between an organoid and the tissue it came from. Integrating artificial intelligence-driven digital pathology with genomic and organoid drug-response data has already been proposed as a route to qualifying PDOs as new approach methodologies in head and neck cancer (76). On the molecular side, unsupervised multi-omics integration frameworks provide a principled way to combine transcriptomic, metabolomic and pharmacological layers from the same organoid line without assuming in advance which layer is dominant (77).

Two cautions apply across all three areas. Models trained on successfully established organoid lines inherit whatever selection bias culture imposes, so a model can be internally accurate and systematically unrepresentative of the patients whose tumors failed to grow. And model performance reported on retrospective cohorts is not evidence of clinical utility. As with the organoid assays themselves, a computational predictor reaches Level 3 only through prospective specification and testing against a predefined outcome.

## 7 From metabolic mechanism to clinical use

### 7.1 Immunometabolism and treatment-induced plasticity

Tumor-organoid co-cultures can support immune-response studies, as established by the generation of tumor-reactive T cells from lymphocyte-organoid co-culture (78) and extended by the colorectal natural killer cell study discussed above (12). For cancer metabolism, the next step is not simply to add immune cells, but to measure the metabolic variable responsible for altered function. Co-culture studies should distinguish oxygen limitation, nutrient competition, inhibitory metabolite accumulation and tumor-intrinsic resistance through paired functional and metabolic readouts.

Metabolic plasticity during treatment also deserves direct testing. Lactate has recently been reported to control cancer stemness and plasticity through epigenetic regulation (79). Organoid studies could evaluate whether this mechanism varies across patient tumors and treatment states, but such experiments require controls for pH, perturbation of lactate production or transport, and readouts of both cell state and treatment response. Until then, lactate-associated organoid therapy resistance should be described as a testable model rather than an established clinical marker.

### 7.2 Functional precision oncology

Recent studies have strengthened the clinical case for patient-derived organoids while also clarifying its limits. Two earlier studies set the template. Organoids from metastatic gastrointestinal cancers reproduced patient responses to the corresponding agents in a prospective co-clinical comparison (80), and pharmacotyping of pancreatic cancer organoids identified subgroups of common chemotherapy responders whose profiles tracked clinical behavior (81). Organoid screens contributed to the development of the EGFR-by-LGR5 bispecific antibody MCLA-158 (82). In metastatic colorectal cancer, a phase 2 study found that organoid-guided sensitivity testing was feasible and that 17 of 34 treated patients remained progression-free at two months (83). A multicenter prospective analysis spanning 17 tumor types later reported variable establishment success and clinicopathological concordance, with treatment responses mirrored in a small longitudinal subset (84).

The prospective OPTIC study offers more recent evidence in metastatic colorectal cancer: organoid response to fluoropyrimidine and oxaliplatin correlated with patient response, with positive and negative predictive values of 0.78 and 0.80 for all target lesions in a prespecified treatment subgroup (85). This is an important advance for functional prediction, but it is not evidence that an organoid captures tumor metabolism comprehensively. Clinical response integrates drug exposure, immunity, stroma and systemic factors that remain incompletely represented in epithelial organoid cultures.

These studies also uncover selection effects relevant to cancer metabolism. Not every biopsy produces a usable organoid, and successful establishment may favor proliferative or culture-adapted cells. A metabolism-driven assay could therefore be informative among successfully cultured specimens while still missing a clinically important subset. Reports should provide establishment success, reasons for failure, time to assay, treatment history and whether metabolic conclusions were verified against tissue not selected by culture.

### 7.3 Barriers to clinical implementation

Feasibility studies and predictive signals do not by themselves create a clinical test. Several barriers separate the studies described above from routine use, and they are worth stating plainly because they determine which validation work is worth doing next.

The first barrier is regulatory. The 2025 announcement by the United States Food and Drug Administration of a plan to reduce, refine or potentially replace selected animal testing requirements using human-relevant approaches has raised the profile of organoids as new approach methodologies (86, 87). That announcement concerns qualification of alternative methods in drug development, and it does not create a regulatory pathway for an organoid assay used to guide treatment of an individual patient. A metabolically motivated organoid assay intended for clinical decision-making would require analytical validation, defined performance characteristics and operation under laboratory accreditation appropriate to the jurisdiction, none of which the current literature reports. Reviews of functional precision oncology and of the clinical applications of human organoids reach the same conclusion from the assay side, that patient-derived functional readouts remain investigational pending prospective qualification (88, 89).

The second barrier is multicenter reproducibility. A multicenter prospective analysis spanning 17 tumor types reported variable establishment success and clinicopathological concordance, with treatment responses mirrored in only a small longitudinal subset (84). Until inter-center variance in establishment, culture and assay conditions is quantified, single-center predictive values cannot be assumed to generalize. This constraint is stronger for metabolic readouts than for cytotoxicity readouts, because metabolic phenotype is more sensitive to culture conditions.

The third barrier is operational. An assay result must arrive within the window in which a treatment decision is made, which in advanced pancreatic or colorectal cancer is typically a few weeks from biopsy. Organoid establishment and expansion to assay-ready biomass frequently exceeds that window, so the realistic near-term application is decision support in second-line and subsequent-line settings rather than first-line selection. Cost and scalability follow from the same constraint, because prolonged culture, specialized media and manual handling dominate per-patient cost, and no formal cost-effectiveness analysis of organoid-guided metabolic decision-making has been published. Implementation also requires a workflow that does not currently exist in most centers, including tissue routing agreements with pathology that preserve diagnostic priority, defined specimen handling, quality-controlled culture facilities and a mechanism for returning a functional result to a multidisciplinary team in a form that can be acted upon (90).

## 8 Limitations of patient-derived organoid models

The preceding sections argue for calibrated claims. That calibration requires an explicit account of what patient-derived organoids currently cannot do, because several of these limitations bear directly on metabolic interpretation rather than being generic caveats.

Establishment success is selective and incomplete. Success rates vary by tumor type, specimen source and prior treatment, and a prospective observational study in pancreatic cancer has begun to identify which clinical and specimen factors predict successful establishment (91). Large biobanking efforts report the same pattern in other tumor types (92). The consequence for cancer metabolism is specific rather than general. If establishment favors proliferative or culture-adapted cells, then metabolic phenotypes that depend on slow cycling, quiescence or stromal support are systematically under-sampled, and the resulting cohort can be internally consistent while remaining unrepresentative of the disease.

Clonal selection and drift occur during culture. Organoid cultures recapitulate substantial tumor heterogeneity and are usable for clonality studies, but they also undergo selection. Single-cell analysis of colorectal cancer organoid systems has shown which cell states are retained and which are lost relative to the source tumor, and esophageal adenocarcinoma organoids have been used explicitly to track clonal dynamics (93, 94). In pancreatic cancer organoids the genomic heterogeneity present at establishment has been reported to remain broadly stable across culture, which usefully separates genomic drift from the phenotypic drift at issue here (95). Because metabolic phenotype is a property of cell state as much as of genotype, a line that remains genomically representative after serial passage may nonetheless have drifted metabolically. Metabolic studies should therefore report passage number and, where a claim is central, demonstrate phenotype stability across passages.

Compartment loss remains the largest structural limitation. Epithelial organoids lack vasculature, and unless deliberately reconstructed they lack stromal, immune and neural compartments. For metabolism this is not a peripheral omission, because perfusion sets nutrient and oxygen delivery, stroma supplies metabolites such as alanine, and immune cells compete for the same nutrients. Systemic influences including diet, endocrine signaling, cachexia and drug pharmacokinetics are absent by construction.

Practical and ethical constraints complete the list. Cost and scalability limit cohort size, which is one reason several of the most informative metabolic studies discussed here involve fewer than a dozen lines. Reproducibility between laboratories is unquantified for metabolic endpoints, as discussed above. Ethical considerations are not resolved by routine consent alone. Organoid biobanking raises questions about secondary use, indefinite propagation of a living patient-derived resource, commercialization and benefit sharing, return of clinically relevant incidental findings, and governance of transfer between institutions and jurisdictions (96). These issues are foreseeable rather than hypothetical, and protocols are best served by addressing them at the point of collection.

## 9 A validation agenda for metabolic organoids

The key unmet need is a matched metabolic fidelity test. For each clinically relevant organoid model, studies should determine whether metabolic states and responses measured in culture correspond to the source tumor under comparable constraints. This requires pairing organoid experiments with source-tumor spatial metabolomics or isotope-resolved data when feasible, rather than relying only on histology, driver genotype or transcriptional similarity. **Figure 3** sets out that pipeline in full, from a single clinical specimen through matched profiling and concordance testing to prospective clinical evaluation, and indicates the evidence level attainable at each stage.

**Figure 3:**
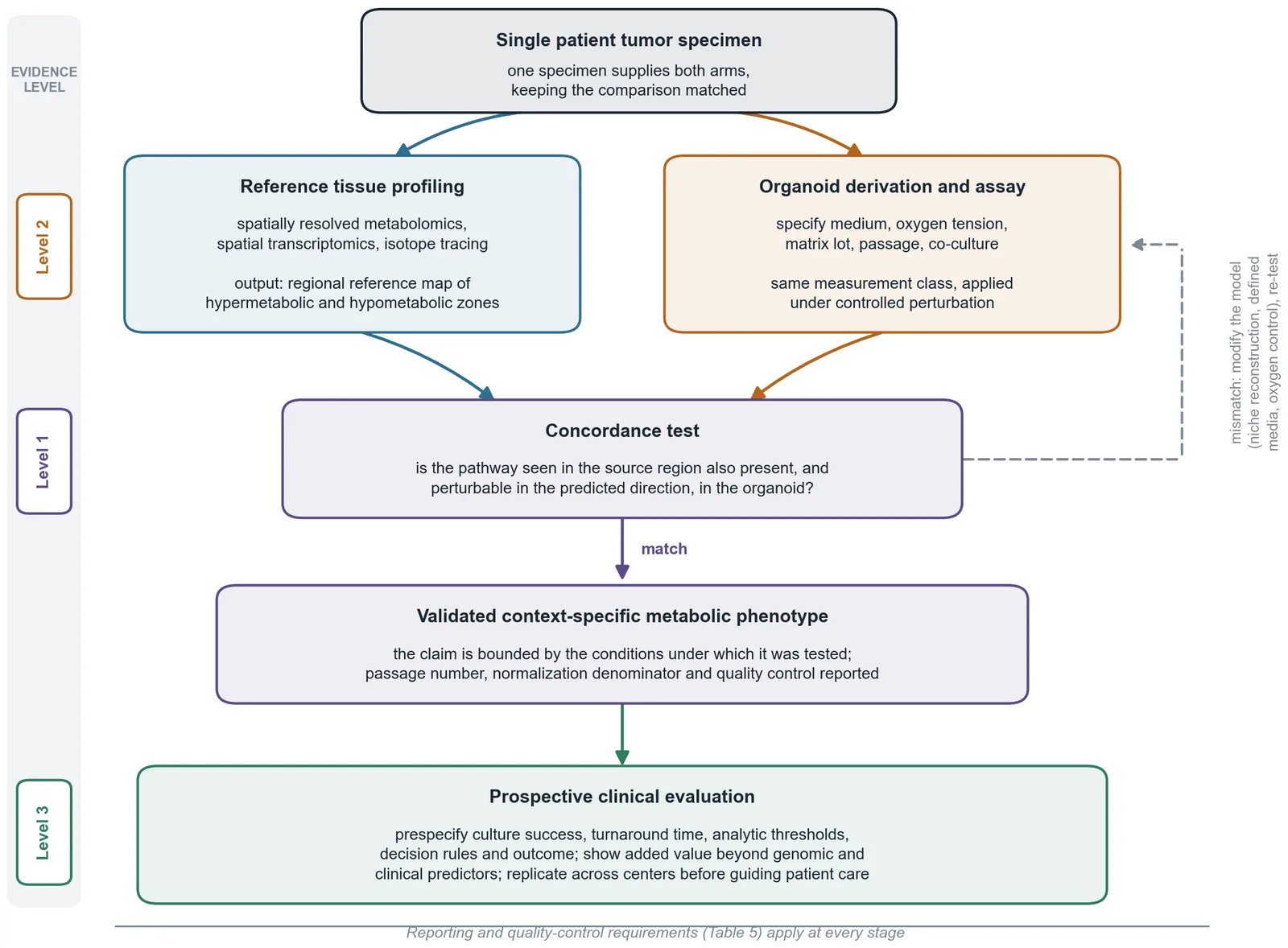
A validation pipeline from patient tissue to clinical application. The schematic sets out the sequence required to convert an organoid metabolic observation into a clinically usable readout. A single clinical specimen supplies both the reference tissue map and the derived organoid, so that the two arms remain matched. Reference profiling uses spatially resolved and isotope-resolved measurement to define the metabolic state of the source tumor by region. The organoid arm specifies medium, oxygen tension, matrix, passage and co-culture composition, then applies the same measurement class under controlled perturbation. Concordance testing compares the two arms for the pathway in question and returns either an accepted match, in which case the phenotype qualifies as Level 1 evidence, or a mismatch, which routes back to model modification such as niche reconstruction. Only phenotypes that pass concordance enter prospective clinical evaluation, where the assay must predefine culture success, turnaround time, analytic thresholds, decision rules and outcome. Quality control and reporting requirements in **Table 5** apply throughout, and the evidence level attainable at each stage is shown on the left.

A second need is experimental specification. Culture matrix, defined media composition, oxygen conditions, organoid dimensions, passage number and co-culture components should be reported because each can modify the phenotype under investigation. Functional conclusions should state whether the endpoint is metabolite abundance, carbon flux, redox state, invasion, immune activity, survival or drug response. An effect on viability alone should not be interpreted as proof of a specific metabolic mechanism.

The minimum analytical standard should depend on the claim. A claim of altered nutrient use requires a flux-sensitive assay or rescue experiment; a claim of spatial heterogeneity requires a spatially resolved measurement; a claim of immune-metabolic suppression requires measurements in both tumor and immune compartments; and a claim of clinical actionability requires a prospectively specified relationship to outcome. This matching of claim to evidence is the most direct safeguard against overstatement.

A third need is model extension guided by evidence rather than by maximum complexity. Stromal, immune, neuronal, vascular or microfluidic elements should be added when they address a defined failure of an epithelial organoid and when their contribution can be quantified. The gastric neuron-organoid model and colorectal natural killer cell co-culture illustrate this principle (11, 12). For systemic influences such as diet, endocrine signals, cachexia or drug pharmacokinetics, organoids will remain one component of a multimodel validation strategy.

Finally, clinical translation demands prospective design. A metabolically motivated organoid assay should predefine culture success, time to result, analytic thresholds, decision rules and outcomes, then show added value beyond genomic and clinical predictors. The recent clinical studies establish feasibility and initial predictive signals (83–85); they also demonstrate why organoid selection can be biased by sample quality and culture success. **Table 5** converts these limitations into testable reporting and validation requirements.

## 10 Conclusions

Tumor organoids are moving cancer metabolism research from plausibility toward direct testing. The most convincing current evidence is concentrated in defined applications: subtype-associated oxidative dependence on mitochondrial pyruvate import and KRAS-linked cholesterol biosynthesis in pancreatic organoids, P4HA1-dependent hypoxia-driven invasion in the same setting, niche-induced dependence on lanosterol synthase and acetyl-CoA carboxylase in gastric organoids, and hypoxia-associated immune programs in colorectal co-cultures. These advances justify greater use of organoids, while also setting a higher standard for claims made in less directly tested tumor settings.

The field’s central gap is no longer the absence of organoid technology; it is the limited demonstration that organoid metabolic phenotypes faithfully reflect spatially heterogeneous patient tumors and predict clinically useful response. Closing that gap will require flux-sensitive measurement, matched tissue benchmarking, purposeful niche reconstruction and prospective clinical evaluation. It will equally require progress on problems that are procedural rather than conceptual, including harmonized analytical quality control and normalization, transparent reporting of culture variables, honest accounting of establishment bias and clonal drift, and computational predictions labeled at the evidence level they actually occupy. Under those conditions, tumor organoids can become rigorous context-specific instruments for cancer metabolism rather than broad surrogates whose validity is presumed.
